# 1,3-Bis(4-bromo­phen­yl)imidazolium chloride dihydrate

**DOI:** 10.1107/S1600536810018581

**Published:** 2010-05-22

**Authors:** Simon J. Garden, Paola E. Gama, Edward R. T. Tiekink, James L. Wardell, Solange M. S. V. Wardell, R. Alan Howie

**Affiliations:** a Instituto de Química, Departamento de Quimica Orgânica, Universidade Federal do Rio de Janeiro, Ilha do Fundão, CT, Bloco A, Rio de Janeiro 21949-900, RJ, Brazil; b Department of Chemistry, University of Malaya, 50603 Kuala Lumpur, Malaysia; c Centro de Desenvolvimento Tecnológico em Saúde (CDTS), Fundação Oswaldo Cruz (FIOCRUZ), Casa Amarela, Campus de Manguinhos, Av. Brasil 4365, 21040-900 Rio de Janeiro, RJ, Brazil; d CHEMSOL, 1 Harcourt Road, Aberdeen AB15 5NY, Scotland; e Department of Chemistry, University of Aberdeen, Old Aberdeen AB15 5NY, Scotland

## Abstract

In the title hydrated salt, C_15_H_11_Br_2_N_2_^+^·Cl^−^·2H_2_O, the complete imidazolium cation is generated by a crystallographic twofold axis, with one C atom lying on the axis. The chloride ion and both water mol­ecules of crystallization also lie on a crystallographic twofold axis of symmetry. The cation is non-planar, the dihedral angle formed between the central imidazolium and benzene rings being 12.9 (3)°; the dihedral angle between the symmetry-related benzene rings is 25.60 (13)°. In the crystal, O—H⋯Cl hydrogen bonds result in supra­molecular chains along *c* mediated by eight-membered {⋯HOH⋯Cl}_2_ synthons. These are consolidated by C—H⋯O and π–π [centroid–centroid distance = 3.687 (3) Å] inter­actions.

## Related literature

For the preparation of imidazolyl­idene carbenes, see: Nolan (2006 ▶); Diez-Gonzalez & Nolan (2007 ▶); Glorius (2007 ▶); Leuthaeusser *et al.* (2007 ▶); Alcarazo *et al.* (2010 ▶). For related structures, see: Luger & Ruban (1975 ▶); Cole & Junk (2004 ▶); Wan *et al.* (2008 ▶).
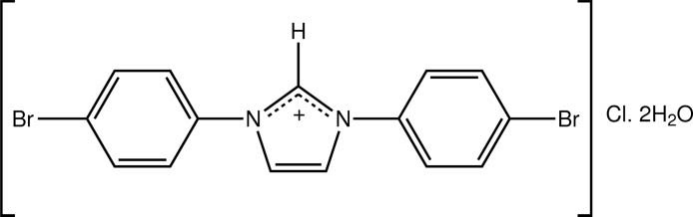


## Experimental

### 

#### Crystal data

C_15_H_11_Br_2_N_2_^+^·Cl^−^·2H_2_O
*M* *_r_* = 450.56
Tetragonal, 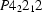

*a* = 17.8377 (7) Å
*c* = 5.1270 (1) Å
*V* = 1631.33 (10) Å^3^
*Z* = 4
Mo *K*α radiation
μ = 5.14 mm^−1^
*T* = 120 K
0.40 × 0.03 × 0.02 mm

#### Data collection

Nonius KappaCCD diffractometer
Absorption correction: multi-scan (*SADABS*; Sheldrick, 2007 ▶) *T*_min_ = 0.665, *T*_max_ = 1.000
13675 measured reflections
1885 independent reflections
1654 reflections with *I* > 2σ(*I*)
*R*_int_ = 0.048

#### Refinement

*R*[*F* ^2^ > 2σ(*F* ^2^)] = 0.032
*wR*(*F* ^2^) = 0.075
*S* = 1.06
1885 reflections
108 parameters
2 restraints
H atoms treated by a mixture of independent and constrained refinement
Δρ_max_ = 0.40 e Å^−3^
Δρ_min_ = −0.69 e Å^−3^
Absolute structure: Flack (1983 ▶), 742 Friedel pairs
Flack parameter: 0.01 (2)

### 

Data collection: *COLLECT* (Hooft, 1998 ▶); cell refinement: *DENZO* (Otwinowski & Minor, 1997 ▶) and *COLLECT*; data reduction: *DENZO* and *COLLECT*; program(s) used to solve structure: *SHELXS97* (Sheldrick, 2008 ▶); program(s) used to refine structure: *SHELXL97* (Sheldrick, 2008 ▶); molecular graphics: *ORTEP-3* (Farrugia, 1997 ▶) and *DIAMOND* (Brandenburg, 2006 ▶); software used to prepare material for publication: *publCIF* (Westrip, 2010 ▶).

## Supplementary Material

Crystal structure: contains datablocks global, I. DOI: 10.1107/S1600536810018581/hb5456sup1.cif

Structure factors: contains datablocks I. DOI: 10.1107/S1600536810018581/hb5456Isup2.hkl

Additional supplementary materials:  crystallographic information; 3D view; checkCIF report

## Figures and Tables

**Table 1:** Hydrogen-bond geometry (Å, °)

*D*—H⋯*A*	*D*—H	H⋯*A*	*D*⋯*A*	*D*—H⋯*A*
O1—H1o⋯Cl1^i^	0.84 (6)	2.28 (6)	3.1116 (19)	170 (8)
O2—H2o⋯Cl1	0.87 (6)	2.40 (6)	3.211 (3)	157 (7)
C1—H1⋯O1	0.95	2.09	3.042 (5)	180
C2—H2⋯O2^ii^	0.95	2.40	3.302 (7)	159

Symmetry codes: (i) 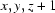; (ii) 
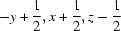
.

**Fig. 1.**
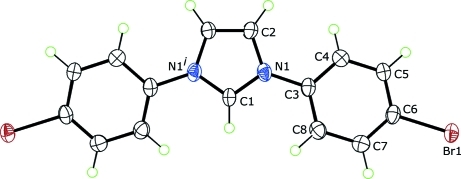
The molecular structure of the cation in (I) showing displacement ellipsoids at the 50% probability level. The C1 atom lies on a two-fold axis. Symmetry operation i: y, x, 2-z.

**Fig. 2.**
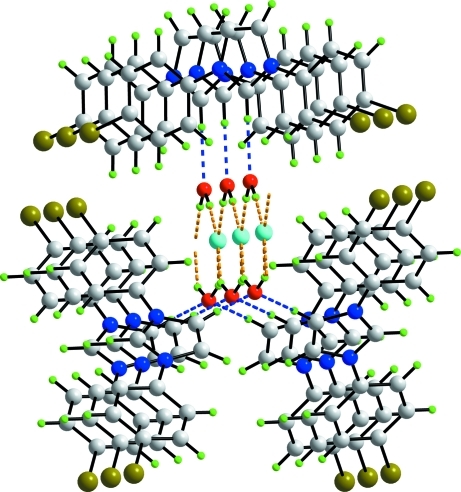
A view highlighting the eight-membered {···HOH···Cl}2 synthons aligned along the c axis in (I). The O–H···O hydrogen bonding and C–H···O interactions are shown as orange and blue dashed lines, respectively. Colour code: Br, olive; O, red; N, blue; C, grey; and H, green.

**Fig. 3.**
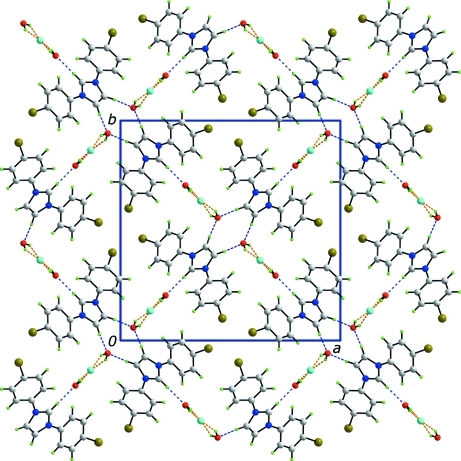
A view in projection down the c axis of the crystal packing in (I). The O–H···O hydrogen bonding and C–H···O interactions are shown as orange and blue dashed lines, respectively. Colour code: Br, olive; O, red; N, blue; C, grey; and H, green.

## supplementary crystallographic information

### Comment

The deprotonation of *N*,*N*-disubstituted imidazolium salts has been extensively used to generate imidazolylidene carbenes for use as ligands for metals or their salts in homogeneous catalysis (Nolan, 2006; Glorius, 2007). The structural motif can be readily varied so as to modify the electronic properties of the carbene and their complexes (Alcarazo *et al.*, 2010; Leuthaeusser *et al.*, 2007; Diez-Gonzalez & Nolan, 2007). Whereas structural studies are available for a number of derivatives (Luger & Ruban, 1975; Cole & Junk, 2004; Wan *et al.*, 2008), little is known about simple 1,3-diphenyl derivatives that do not posses substituents in the 2,6-positions of the phenyl rings. As part of a study into structural effects of these carbenes, we have been able to prepare and crystallize for the first time the salt 1,3-di-(4-bromophenyl)imidazolium chloride, isolated as a dihydrate, (I).

The crystallographic asymmetric unit of (I) comprises half a 1,3-di-(4-bromophenyl)imidazolium cation, Fig. 1, half a chloride, and two half water molecules, as each of the aforementioned species lies on a two-fold axis of symmetry. The cation is non-planar with the dihedral angle formed between the central imidazolium ring [r.m.s. deviation = 0.005 Å] and the benzene ring (C3–C8) being 12.9 (3) °; the dihedral angle formed between the symmetry related benzene rings is 25.60 (13) °. The twists between the rings allows for the close approach of a water molecule allowing the formation of a C1—H···O1 interaction, Table 1. This O1-water molecule also forms O–H···Cl interactions with the chloride which in turn is connected to the second water molecule leading to eight-membered {···HOH···Cl}_2_ synthons aligned along the *c* axis, Fig. 2 and Table 1. The three-dimensional packing is consolidated by further C–H···O2 interactions, Fig. 3, as well as π–π contacts (along *c*) between the imidazolium and between rings [ring centroid···ring centroid distance = 3.687 (3) Å, angle of inclination = 12.9 (3) ° for *i*: *x*, *y*, 1+*z*].

### Experimental

*p*-Bromoaniline (50 mmol) was solubilised in AcOH/H_2_O (3:1 V/V, 40 ml). Aqueous formaldehyde (37%, 2 ml) was added to the solution resulting in the precipitation of a solid. Following this, aqueous glyoxal (40%, 3 ml) was added and the reaction mixture was subsequently warmed (333 K) for 30 minutes. Finally, aqueous HCl (3M, 10 ml) was added resulting in the formation of a homogeneous solution. Heating was continued for a further 30 min. The crude product was precipitated from the reaction by diluting with water. The solid was isolated by filtration and allowed to air dry. The product was recrystallized from 2-propanol to generate colourless needles of (I). Melting point 581–583 K; 83% yield. ^1^H NMR (DMSO-d_6_/CDCl_3_): δ 10.48 [1H, s]; 8.55 [2H, s]; 7.90 [4H, s] p.p.m. ^13^C NMR (DMSO-d_6_/CDCl_3_): δ 122.1; 123.2; 124.3; 133.2; 134.0; 135.0 p.p.m. IR (cm^-1^): 3365, 3092, 3048, 1556, 1488, 1309, 1259, 1075, 1008, 824.

### Refinement

The C-bound H atoms were geometrically placed (C–H = 0.95 Å) and refined as riding with *U*_iso_(H) = 1.2*U*_eq_(C). The water-bound H atoms were refined with O–H = 0.84±0.01 Å, and with *U*_iso_(H) = 1.5*U*_eq_(O).

### Crystal data

**Table:**

C_15_H_11_Br_2_N_2_^+^·Cl^−^·2H_2_O	*D*_x_ = 1.835 Mg m^−^^3^
*M**_r_* = 450.56	Mo *K*α radiation, λ = 0.71073 Å
Tetragonal, *P*4_2_2_1_2	Cell parameters from 2084 reflections
Hall symbol: P 4n 2n	θ = 2.9–27.5°
*a* = 17.8377 (7) Å	µ = 5.14 mm^−^^1^
*c* = 5.1270 (1) Å	*T* = 120 K
*V* = 1631.33 (10) Å^3^	Needle, colourless
*Z* = 4	0.40 × 0.03 × 0.02 mm
*F*(000) = 888	

### Data collection

**Table:**

Nonius KappaCCD diffractometer	1885 independent reflections
Radiation source: Enraf Nonius FR591 rotating anode	1654 reflections with *I* > 2σ(*I*)
10 cm confocal mirrors	*R*_int_ = 0.048
Detector resolution: 9.091 pixels mm^-1^	θ_max_ = 27.5°, θ_min_ = 3.2°
φ and ω scans	*h* = −23→23
Absorption correction: multi-scan (*SADABS*; Sheldrick, 2007)	*k* = −14→23
*T*_min_ = 0.665, *T*_max_ = 1.000	*l* = −6→6
13675 measured reflections	

### Refinement

**Table:**

Refinement on *F*^2^	Secondary atom site location: difference Fourier map
Least-squares matrix: full	Hydrogen site location: inferred from neighbouring sites
*R*[*F*^2^ > 2σ(*F*^2^)] = 0.032	H atoms treated by a mixture of independent and constrained refinement
*wR*(*F*^2^) = 0.075	*w* = 1/[σ^2^(*F*_o_^2^) + (0.0285*P*)^2^ + 2.7306*P*] where *P* = (*F*_o_^2^ + 2*F*_c_^2^)/3
*S* = 1.06	(Δ/σ)_max_ = 0.001
1885 reflections	Δρ_max_ = 0.40 e Å^−^^3^
108 parameters	Δρ_min_ = −0.69 e Å^−^^3^
2 restraints	Absolute structure: Flack (1983), 742 Friedel pairs
Primary atom site location: structure-invariant direct methods	Flack parameter: 0.01 (2)

### Special details

**Table:**

Geometry. All s.u.'s (except the s.u. in the dihedral angle between two l.s. planes) are estimated using the full covariance matrix. The cell s.u.'s are taken into account individually in the estimation of s.u.'s in distances, angles and torsion angles; correlations between s.u.'s in cell parameters are only used when they are defined by crystal symmetry. An approximate (isotropic) treatment of cell s.u.'s is used for estimating s.u.'s involving l.s. planes.
Refinement. Refinement of *F*^2^ against ALL reflections. The weighted *R*-factor wR and goodness of fit *S* are based on *F*^2^, conventional *R*-factors *R* are based on *F*, with *F* set to zero for negative *F*^2^. The threshold expression of *F*^2^ > 2σ(*F*^2^) is used only for calculating *R*-factors(gt) etc. and is not relevant to the choice of reflections for refinement. *R*-factors based on *F*^2^ are statistically about twice as large as those based on *F*, and *R*- factors based on ALL data will be even larger.

### Fractional atomic coordinates and isotropic or equivalent isotropic displacement parameters (Å^2^)

**Table:**

	*x*	*y*	*z*	*U*_iso_*/*U*_eq_	
Br1	0.100493 (18)	0.463088 (18)	0.00124 (12)	0.02761 (11)	
Cl1	0.13502 (7)	0.13502 (7)	0.5000	0.0552 (4)	
O1	0.20493 (17)	0.20493 (17)	1.0000	0.0681 (15)	
H1O	0.186 (5)	0.181 (5)	1.125 (8)	0.102*	
O2	0.0584 (3)	0.0584 (3)	1.0000	0.0794 (16)	
H2O	0.067 (5)	0.087 (4)	0.867 (10)	0.119*	
N1	0.32639 (19)	0.38584 (18)	0.8472 (7)	0.0274 (7)	
C1	0.32553 (19)	0.32553 (19)	1.0000	0.0270 (9)	
H1	0.2879	0.2879	1.0000	0.032*	
C2	0.3893 (3)	0.4253 (3)	0.9090 (14)	0.083 (3)	
H2	0.4044	0.4714	0.8322	0.100*	
C3	0.2714 (2)	0.4041 (2)	0.6525 (7)	0.0252 (8)	
C4	0.2720 (2)	0.4748 (2)	0.5411 (11)	0.0382 (13)	
H4	0.3075	0.5111	0.5974	0.046*	
C5	0.2207 (3)	0.4925 (3)	0.3464 (9)	0.0369 (10)	
H5	0.2209	0.5408	0.2685	0.044*	
C6	0.1696 (2)	0.4391 (2)	0.2680 (7)	0.0267 (9)	
C7	0.1683 (2)	0.3694 (2)	0.3799 (8)	0.0265 (9)	
H7	0.1325	0.3334	0.3241	0.032*	
C8	0.2194 (2)	0.3512 (2)	0.5749 (8)	0.0289 (10)	
H8	0.2185	0.3030	0.6536	0.035*	

### Atomic displacement parameters (Å^2^)

**Table:**

	*U*^11^	*U*^22^	*U*^33^	*U*^12^	*U*^13^	*U*^23^
Br1	0.02725 (18)	0.0350 (2)	0.02061 (17)	0.00424 (13)	−0.0019 (3)	0.0025 (3)
Cl1	0.0652 (6)	0.0652 (6)	0.0352 (8)	−0.0031 (8)	0.0037 (10)	−0.0037 (10)
O1	0.079 (2)	0.079 (2)	0.046 (3)	−0.049 (3)	−0.009 (4)	0.009 (4)
O2	0.080 (2)	0.080 (2)	0.078 (4)	0.008 (3)	0.018 (4)	−0.018 (4)
N1	0.0252 (17)	0.0274 (18)	0.0296 (19)	0.0075 (14)	−0.0003 (15)	0.0059 (15)
C1	0.0298 (14)	0.0298 (14)	0.021 (2)	0.0017 (19)	−0.005 (3)	0.005 (3)
C2	0.056 (3)	0.061 (3)	0.132 (7)	−0.030 (3)	−0.064 (4)	0.064 (4)
C3	0.0245 (19)	0.030 (2)	0.021 (2)	0.0066 (16)	0.0005 (15)	0.0017 (16)
C4	0.038 (2)	0.033 (2)	0.044 (4)	−0.0073 (16)	−0.011 (2)	0.011 (2)
C5	0.038 (2)	0.033 (2)	0.039 (3)	−0.0040 (19)	−0.013 (2)	0.014 (2)
C6	0.0221 (19)	0.036 (2)	0.0219 (19)	0.0067 (16)	0.0002 (15)	0.0025 (16)
C7	0.0219 (19)	0.027 (2)	0.030 (2)	0.0039 (16)	0.0048 (17)	−0.0021 (17)
C8	0.026 (2)	0.029 (2)	0.032 (3)	0.0052 (16)	0.0026 (15)	0.0037 (15)

### Geometric parameters (Å, °)

**Table:**

Br1—C6	1.890 (4)	C3—C8	1.383 (6)
O1—H1O	0.84 (6)	C3—C4	1.383 (6)
O2—H2O	0.87 (6)	C4—C5	1.391 (6)
N1—C1	1.331 (4)	C4—H4	0.9500
N1—C2	1.362 (6)	C5—C6	1.378 (6)
N1—C3	1.437 (5)	C5—H5	0.9500
C1—N1^i^	1.331 (4)	C6—C7	1.370 (6)
C1—H1	0.9500	C7—C8	1.391 (6)
C2—C2^i^	1.303 (10)	C7—H7	0.9500
C2—H2	0.9500	C8—H8	0.9500
			
C1—N1—C2	106.9 (4)	C5—C4—H4	120.1
C1—N1—C3	125.8 (4)	C6—C5—C4	119.2 (4)
C2—N1—C3	127.3 (4)	C6—C5—H5	120.4
N1—C1—N1^i^	109.1 (5)	C4—C5—H5	120.4
N1—C1—H1	125.4	C7—C6—C5	121.1 (4)
N1^i^—C1—H1	125.4	C7—C6—Br1	119.8 (3)
C2^i^—C2—N1	108.5 (3)	C5—C6—Br1	119.1 (3)
C2^i^—C2—H2	125.7	C6—C7—C8	120.2 (4)
N1—C2—H2	125.7	C6—C7—H7	119.9
C8—C3—C4	120.6 (4)	C8—C7—H7	119.9
C8—C3—N1	120.2 (4)	C3—C8—C7	119.1 (4)
C4—C3—N1	119.2 (4)	C3—C8—H8	120.5
C3—C4—C5	119.9 (4)	C7—C8—H8	120.5
C3—C4—H4	120.1		
			
C2—N1—C1—N1^i^	−0.2 (4)	N1—C3—C4—C5	177.9 (4)
C3—N1—C1—N1^i^	178.9 (4)	C3—C4—C5—C6	0.2 (7)
C1—N1—C2—C2^i^	0.7 (10)	C4—C5—C6—C7	0.5 (7)
C3—N1—C2—C2^i^	−178.4 (6)	C4—C5—C6—Br1	−179.4 (4)
C1—N1—C3—C8	−12.5 (5)	C5—C6—C7—C8	−0.4 (6)
C2—N1—C3—C8	166.4 (5)	Br1—C6—C7—C8	179.5 (3)
C1—N1—C3—C4	168.5 (4)	C4—C3—C8—C7	1.0 (6)
C2—N1—C3—C4	−12.5 (7)	N1—C3—C8—C7	−177.9 (3)
C8—C3—C4—C5	−1.0 (7)	C6—C7—C8—C3	−0.3 (6)

Symmetry codes: (i) *y*, *x*, −*z*+2.

### Hydrogen-bond geometry (Å, °)

**Table:**

*D*—H···*A*	*D*—H	H···*A*	*D*···*A*	*D*—H···*A*
O1—H1o···Cl1^ii^	0.84 (6)	2.28 (6)	3.1116 (19)	170 (8)
O2—H2o···Cl1	0.87 (6)	2.40 (6)	3.211 (3)	157 (7)
C1—H1···O1	0.95	2.09	3.042 (5)	180
C2—H2···O2^iii^	0.95	2.40	3.302 (7)	159

Symmetry codes: (ii) *x*, *y*, *z*+1;  (iii) −*y*+1/2, *x*+1/2, *z*−1/2.
